# Poly[(μ-2-acet­oxy­benzoato)(2-acet­oxy­benzoato)-μ-aqua-mercury(II)]

**DOI:** 10.1107/S1600536811029278

**Published:** 2011-07-30

**Authors:** J. PrakashaReddy

**Affiliations:** a Department of Chemistry, MS015, Brandeis University, Waltham, MA 02454, USA

## Abstract

In the title compound, [Hg(C_9_H_7_O_4_)_2_(H_2_O)]_*n*_, the Hg^II^ ion is five-coordinated by three acetylsalicylate anions and water leading to the formation of a coordination polymer extending parallel to (001). O—H⋯O and C—H⋯O hydrogen bonds are effective in the stabilization of the crystal structure.

## Related literature

For general background to metal complexes with acetyl­salicylate as a ligand, see: Manojlović-Muir (1973 ▶); Garcia *et al.* (2003 ▶); Greenaway *et al.* (1984 ▶); Fujimori *et al.* (2005 ▶); James *et al.* (1998 ▶); Vasquez-Arciga *et al.* (2004) ▶; Ma & Moulton (2007 ▶). 
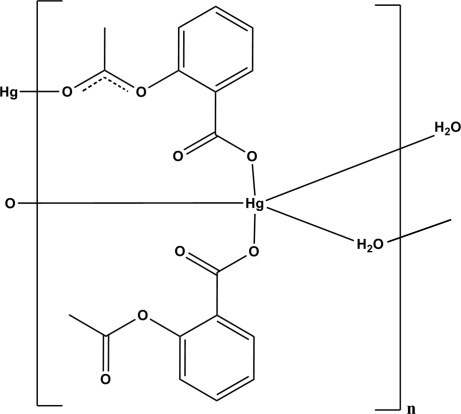


## Experimental

### 

#### Crystal data

[Hg(C_9_H_7_O_4_)_2_(H_2_O)]
*M* *_r_* = 1153.80
Triclinic, 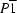

*a* = 6.1851 (9) Å
*b* = 10.1359 (17) Å
*c* = 15.453 (2) Å
α = 100.308 (7)°
β = 98.700 (8)°
γ = 100.667 (7)°
*V* = 919.5 (2) Å^3^
*Z* = 1
Mo *K*α radiation
μ = 8.42 mm^−1^
*T* = 120 K
0.21 × 0.17 × 0.02 mm

#### Data collection

Bruker Kappa APEXII diffractometer
Absorption correction: analytical (De Meulenaer & Tompa, 1965 ▶) *T*_min_ = 0.24, *T*_max_ = 0.83
9510 measured reflections
5293 independent reflections
4404 reflections with *I* > 2σ(*I*)
*R*_int_ = 0.037

#### Refinement

*R*[*F* ^2^ > 2σ(*F* ^2^)] = 0.040
*wR*(*F* ^2^) = 0.092
*S* = 0.97
5293 reflections
253 parameters
H-atom parameters constrained
Δρ_max_ = 1.88 e Å^−3^
Δρ_min_ = −2.19 e Å^−3^

### 

Data collection: *APEX2* (Bruker, 2006 ▶); cell refinement: *SAINT* (Bruker, 2006 ▶); data reduction: *SAINT*; program(s) used to solve structure: *SIR92* (Altomare *et al.*, 1994 ▶); program(s) used to refine structure: *CRYSTALS* (Betteridge *et al.*, 2003 ▶); molecular graphics: *Mercury* (Macrae *et al.*, 2006 ▶); software used to prepare material for publication: *CRYSTALS*.

## Supplementary Material

Crystal structure: contains datablock(s) global, I. DOI: 10.1107/S1600536811029278/dn2701sup1.cif

Structure factors: contains datablock(s) I. DOI: 10.1107/S1600536811029278/dn2701Isup2.hkl

Additional supplementary materials:  crystallographic information; 3D view; checkCIF report

## Figures and Tables

**Table 1:** Hydrogen-bond geometry (Å, °)

*D*—H⋯*A*	*D*—H	H⋯*A*	*D*⋯*A*	*D*—H⋯*A*
O9—H2⋯O2^i^	0.82	2.01	2.835 (7)	180 (1)
O9—H1⋯O5^i^	0.82	1.93	2.758 (5)	179 (1)
C9—H92⋯O1^ii^	0.95	2.49	3.409 (9)	163
C15—H151⋯O4^iii^	0.95	2.51	3.221 (8)	132
C18—H181⋯O5^iii^	0.95	2.59	3.530 (9)	169

Symmetry codes: (i) 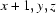; (ii) 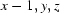; (iii) 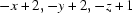.

**Fig. 1.**
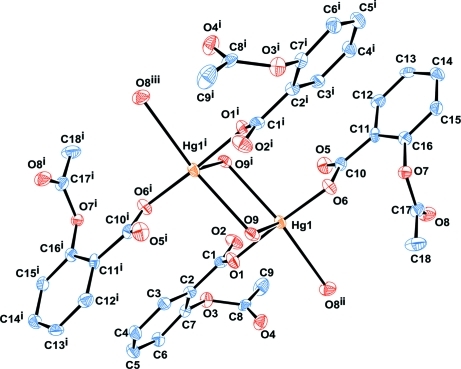
The title compound with the atom labeling scheme. Displacement ellipsoids are drawn at the 50% probability level. H atoms have been omitted for clarity. [Symmetry codes: (i) 2 - x, 1 - y, 1 - z; (ii) 2 - x, 2 - y, 1 - z; (iii) 1 + x,y,z

**Fig. 2.**
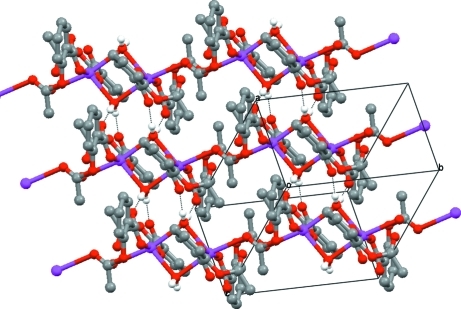
Packing diagram of the title compound. Hydrogen-bond interactions are drawn with dashed lines. H atoms not involved in hydrogen bondings have been omitted for clarity.

## supplementary crystallographic information

### Comment

First crystal structure of copper and acetylsalicylic acid (ASA) metal complex was reported by Manojlović-Muir (1973) which was reinvestigated by Garcia *et al.* (2003). Various other metal complexes of ASA have been also reported (Greenaway *et al.*, 1984; Fujimori *et al.*, 2005; James *et al.*, 1998; Ma & Moulton, 2007; Vásquez-Árciga *et al.*, 2004). For further investigation of the ASA complexes, we synthesized the title compound.

The asymmetric unit of the title compound contains two molecules of ASA, and a bridging water molecule coordinated to the Hg^II^ ion. The molecular arrangement around Hg^II^ is shown in Fig. 1. Crystal structure analysis shows that the carboxylate of the ASA molecules interacts with Hg^II^ in a monodentate fashion while acetyl group O atom of one of the ASA molecules coordinated to Hg^II^ ion forming a coordination polymer. The uncoordinated O atom of the carboxylate form intermolecular O—H···O hydrogen bonds with bridging water molecule resulting in the build up of a two-dimensional network parallel to the (001) plane (Fig. 2 and Table 1). Within this layer exist also weak C—H···O interactions (Table 1).

### Experimental

The title compound was synthesized by adding a solution of acetylsalicylic acid (36 mg, 0.2 mmol) dissolved in methanol (5 ml) to a mercuric chloride (27 mg, 0.1 mmol) dissolved in methanol (5 ml) in a 2:1 ratio respectively. After 3–4 days, colourless crystals of the title compound were obtained on slow evaporation of the solvent.

### Refinement

H atoms bound to the C and O were positioned geometrically and refined as riding atoms,with respective C—H and O—H distances of 0.95 Å and 0.82 Å and with *U*_iso_(H) = 1.2Ueq(C, N).

### Crystal data

**Table:**

[Hg(C_9_H_7_O_4_)_2_(H_2_O)]	*Z* = 1
*M**_r_* = 1153.80	*F*(000) = 552
Triclinic, *P*1	*D*_x_ = 2.084 Mg m^−^^3^
Hall symbol: -P 1	Mo *K*α radiation, λ = 0.71073 Å
*a* = 6.1851 (9) Å	Cell parameters from 3872 reflections
*b* = 10.1359 (17) Å	θ = 3–30°
*c* = 15.453 (2) Å	µ = 8.42 mm^−^^1^
α = 100.308 (7)°	*T* = 120 K
β = 98.700 (8)°	Plate, colorless
γ = 100.667 (7)°	0.21 × 0.17 × 0.02 mm
*V* = 919.5 (2) Å^3^	

### Data collection

**Table:**

Bruker Kappa APEXII diffractometer	4404 reflections with *I* > 2σ(*I*)
graphite	*R*_int_ = 0.037
φ & ω scans	θ_max_ = 30.0°, θ_min_ = 2.2°
Absorption correction: analytical (De Meulenaer & Tompa, 1965)	*h* = −8→7
*T*_min_ = 0.24, *T*_max_ = 0.83	*k* = −14→13
9510 measured reflections	*l* = −21→21
5293 independent reflections	

### Refinement

**Table:**

Refinement on *F*^2^	Primary atom site location: structure-invariant direct methods
Least-squares matrix: full	Hydrogen site location: inferred from neighbouring sites
*R*[*F*^2^ > 2σ(*F*^2^)] = 0.040	H-atom parameters constrained
*wR*(*F*^2^) = 0.092	Method = Modified Sheldrick *w* = 1/[σ^2^(*F*^2^) + (0.04*P*)^2^ + 6.15*P*], where *P* = [max(*F*_o_^2^,0) + 2*F*_c_^2^]/3
*S* = 0.97	(Δ/σ)_max_ = 0.001
5293 reflections	Δρ_max_ = 1.88 e Å^−^^3^
253 parameters	Δρ_min_ = −2.19 e Å^−^^3^
0 restraints	

### Fractional atomic coordinates and isotropic or equivalent isotropic displacement parameters (Å^2^)

**Table:**

	*x*	*y*	*z*	*U*_iso_*/*U*_eq_	
Hg1	0.89515 (4)	0.65817 (2)	0.467447 (16)	0.0168	
O1	0.8495 (8)	0.5346 (4)	0.3440 (3)	0.0248	
O2	0.4800 (8)	0.5246 (5)	0.3271 (3)	0.0285	
O3	0.2895 (7)	0.4772 (4)	0.1441 (3)	0.0215	
O4	0.5203 (8)	0.6855 (4)	0.1796 (4)	0.0281	
O5	0.6204 (8)	0.7673 (5)	0.5843 (3)	0.0270	
O6	0.9879 (7)	0.7872 (4)	0.5911 (3)	0.0222	
O7	1.2498 (7)	1.0048 (4)	0.7117 (3)	0.0174	
O8	1.0778 (8)	1.1274 (5)	0.6272 (3)	0.0268	
O9	1.2672 (7)	0.5721 (4)	0.4763 (3)	0.0198	
C1	0.6439 (11)	0.4964 (6)	0.2984 (4)	0.0198	
C2	0.6323 (10)	0.4156 (6)	0.2068 (4)	0.0165	
C3	0.7982 (11)	0.3423 (6)	0.1913 (4)	0.0205	
C4	0.7977 (11)	0.2696 (6)	0.1066 (5)	0.0231	
C5	0.6320 (11)	0.2710 (6)	0.0339 (5)	0.0249	
C6	0.4648 (11)	0.3435 (6)	0.0472 (4)	0.0216	
C7	0.4668 (10)	0.4133 (6)	0.1336 (4)	0.0178	
C8	0.3385 (11)	0.6172 (6)	0.1734 (4)	0.0207	
C9	0.1367 (13)	0.6643 (7)	0.1952 (6)	0.0365	
C10	0.8163 (11)	0.8122 (6)	0.6245 (4)	0.0195	
C11	0.8677 (10)	0.8985 (5)	0.7175 (4)	0.0171	
C12	0.7009 (11)	0.8886 (6)	0.7696 (5)	0.0223	
C13	0.7399 (12)	0.9606 (6)	0.8567 (4)	0.0238	
C14	0.9507 (11)	1.0455 (6)	0.8963 (4)	0.0227	
C15	1.1151 (11)	1.0597 (6)	0.8456 (4)	0.0204	
C16	1.0744 (10)	0.9876 (6)	0.7573 (4)	0.0174	
C17	1.2367 (11)	1.0780 (6)	0.6470 (4)	0.0224	
C18	1.4387 (13)	1.0861 (8)	0.6063 (5)	0.0333	
H31	0.9129	0.3424	0.2398	0.0255*	
H41	0.9097	0.2185	0.0975	0.0281*	
H51	0.6341	0.2225	−0.0244	0.0287*	
H61	0.3524	0.3452	−0.0016	0.0251*	
H91	0.1725	0.7613	0.2154	0.0468*	
H92	0.0862	0.6224	0.2410	0.0468*	
H93	0.0218	0.6394	0.1432	0.0468*	
H121	0.5576	0.8309	0.7441	0.0281*	
H131	0.6235	0.9526	0.8904	0.0321*	
H141	0.9795	1.0926	0.9572	0.0282*	
H151	1.2569	1.1192	0.8710	0.0242*	
H181	1.4269	1.1379	0.5610	0.0441*	
H182	1.5677	1.1294	0.6511	0.0441*	
H183	1.4512	0.9960	0.5807	0.0441*	
H2	1.3284	0.5582	0.4330	0.0237*	
H1	1.3720	0.6302	0.5086	0.0237*	

### Atomic displacement parameters (Å^2^)

**Table:**

	*U*^11^	*U*^22^	*U*^33^	*U*^12^	*U*^13^	*U*^23^
Hg1	0.01839 (11)	0.01710 (10)	0.01515 (12)	0.00792 (7)	0.00211 (8)	0.00097 (7)
O1	0.028 (2)	0.025 (2)	0.021 (2)	0.0116 (18)	0.0052 (19)	−0.0016 (18)
O2	0.027 (3)	0.042 (3)	0.022 (2)	0.018 (2)	0.007 (2)	0.005 (2)
O3	0.015 (2)	0.022 (2)	0.028 (3)	0.0090 (16)	0.0011 (18)	0.0050 (18)
O4	0.026 (2)	0.021 (2)	0.040 (3)	0.0077 (18)	0.012 (2)	0.009 (2)
O5	0.024 (2)	0.029 (2)	0.026 (3)	0.0072 (18)	−0.002 (2)	0.0028 (19)
O6	0.022 (2)	0.025 (2)	0.017 (2)	0.0076 (17)	0.0018 (18)	−0.0020 (17)
O7	0.020 (2)	0.0172 (18)	0.019 (2)	0.0097 (15)	0.0057 (17)	0.0068 (16)
O8	0.032 (3)	0.029 (2)	0.027 (3)	0.0148 (19)	0.008 (2)	0.015 (2)
O9	0.021 (2)	0.023 (2)	0.014 (2)	0.0036 (16)	0.0030 (17)	0.0014 (16)
C1	0.025 (3)	0.020 (3)	0.017 (3)	0.007 (2)	0.005 (2)	0.008 (2)
C2	0.014 (3)	0.019 (3)	0.018 (3)	0.005 (2)	0.002 (2)	0.006 (2)
C3	0.026 (3)	0.020 (3)	0.018 (3)	0.009 (2)	0.004 (3)	0.007 (2)
C4	0.024 (3)	0.020 (3)	0.026 (4)	0.008 (2)	0.009 (3)	0.002 (2)
C5	0.029 (3)	0.024 (3)	0.020 (3)	0.004 (2)	0.005 (3)	−0.001 (2)
C6	0.022 (3)	0.022 (3)	0.019 (3)	0.005 (2)	−0.001 (2)	0.003 (2)
C7	0.016 (3)	0.015 (2)	0.022 (3)	0.003 (2)	0.002 (2)	0.004 (2)
C8	0.025 (3)	0.019 (3)	0.022 (3)	0.009 (2)	0.006 (3)	0.012 (2)
C9	0.031 (4)	0.029 (3)	0.056 (5)	0.015 (3)	0.013 (4)	0.016 (3)
C10	0.025 (3)	0.015 (2)	0.021 (3)	0.008 (2)	0.005 (2)	0.005 (2)
C11	0.017 (3)	0.012 (2)	0.021 (3)	0.006 (2)	−0.001 (2)	0.001 (2)
C12	0.021 (3)	0.022 (3)	0.027 (4)	0.006 (2)	0.008 (3)	0.009 (3)
C13	0.034 (4)	0.026 (3)	0.020 (3)	0.015 (3)	0.014 (3)	0.013 (3)
C14	0.032 (4)	0.023 (3)	0.016 (3)	0.013 (3)	0.003 (3)	0.004 (2)
C15	0.024 (3)	0.016 (2)	0.021 (3)	0.005 (2)	0.002 (2)	0.003 (2)
C16	0.019 (3)	0.018 (2)	0.019 (3)	0.011 (2)	0.003 (2)	0.005 (2)
C17	0.028 (3)	0.018 (3)	0.023 (3)	0.007 (2)	0.003 (3)	0.007 (2)
C18	0.036 (4)	0.041 (4)	0.034 (4)	0.013 (3)	0.019 (3)	0.020 (3)

### Geometric parameters (Å, °)

**Table:**

Hg1—O9^i^	2.708 (4)	C5—C6	1.394 (9)
Hg1—O8^ii^	2.823 (4)	C5—H51	0.950
Hg1—O1	2.034 (4)	C6—C7	1.393 (9)
Hg1—O6	2.046 (4)	C6—H61	0.950
Hg1—O9	2.601 (4)	C8—C9	1.479 (10)
O1—C1	1.309 (8)	C9—H91	0.950
O2—C1	1.226 (8)	C9—H92	0.950
O3—C7	1.388 (7)	C9—H93	0.950
O3—C8	1.372 (7)	C10—C11	1.497 (8)
O4—C8	1.187 (8)	C11—C12	1.402 (9)
O5—C10	1.236 (8)	C11—C16	1.401 (8)
O6—C10	1.295 (7)	C12—C13	1.373 (9)
O7—C16	1.382 (7)	C12—H121	0.950
O7—C17	1.348 (8)	C13—C14	1.403 (9)
O8—C17	1.207 (8)	C13—H131	0.950
O9—H2	0.820	C14—C15	1.377 (9)
O9—H1	0.820	C14—H141	0.950
C1—C2	1.488 (9)	C15—C16	1.389 (9)
C2—C3	1.398 (8)	C15—H151	0.950
C2—C7	1.400 (8)	C17—C18	1.477 (10)
C3—C4	1.382 (9)	C18—H181	0.950
C3—H31	0.950	C18—H182	0.950
C4—C5	1.406 (10)	C18—H183	0.950
C4—H41	0.950		
			
O9—Hg1—O8	107.12 (11)	O4—C8—O3	121.8 (5)
O9—Hg1—O6	94.68 (16)	O4—C8—C9	127.9 (5)
O1—Hg1—O6	171.97 (18)	O3—C8—C9	110.3 (5)
O1—Hg1—O9	78.28 (14)	O5—C10—C11	121.1 (5)
C1—O1—Hg1	116.6 (3)	O6—C10—C11	116.1 (5)
C8—O3—C7	118.1 (4)	C12—C11—C16	117.2 (5)
C17—O7—C16	117.7 (4)	C12—C11—C10	118.4 (5)
O2—C1—O1	124.4 (5)	C16—C11—C10	124.4 (5)
O2—C1—C2	123.5 (5)	C13—C12—C11	121.9 (5)
O1—C1—C2	112.1 (5)	C12—C13—C14	120.1 (5)
C3—C2—C7	117.5 (5)	C15—C14—C13	118.9 (5)
C3—C2—C1	119.1 (5)	C16—C15—C14	120.2 (5)
C7—C2—C1	123.4 (5)	C15—C16—O7	116.4 (5)
C4—C3—C2	121.0 (5)	C15—C16—C11	121.6 (5)
C5—C4—C3	120.1 (5)	O7—C16—C11	122.0 (5)
C4—C5—C6	120.4 (5)	O8—C17—O7	123.1 (5)
C7—C6—C5	118.6 (5)	O8—C17—C18	126.5 (6)
C6—C7—O3	116.2 (5)	O7—C17—C18	110.4 (5)
C6—C7—C2	122.4 (5)	H181—C18—H183	110.00
O3—C7—C2	121.3 (5)	H182—C18—H183	109.00
			
O9—Hg1—O1—C1	163.5 (4)	C7—O3—C8—O4	9.0 (8)
O9—Hg1—O6—C10	−156.3 (3)	C7—O3—C8—C9	−170.8 (5)
Hg1—O1—C1—O2	−3.7 (7)	Hg1—O6—C10—O5	−3.0 (6)
Hg1—O1—C1—C2	175.2 (3)	Hg1—O6—C10—C11	176.3 (3)
O2—C1—C2—C3	−154.5 (5)	O5—C10—C11—C12	22.0 (8)
O1—C1—C2—C3	26.5 (7)	O6—C10—C11—C12	−157.4 (5)
O2—C1—C2—C7	27.9 (8)	O5—C10—C11—C16	−159.3 (5)
O1—C1—C2—C7	−151.1 (5)	O6—C10—C11—C16	21.3 (7)
C7—C2—C3—C4	−0.3 (8)	C16—C11—C12—C13	−1.9 (8)
C1—C2—C3—C4	−178.0 (5)	C10—C11—C12—C13	176.9 (5)
C2—C3—C4—C5	1.1 (8)	C11—C12—C13—C14	−0.3 (8)
C3—C4—C5—C6	−0.6 (8)	C12—C13—C14—C15	2.8 (8)
C4—C5—C6—C7	−0.5 (8)	C14—C15—C16—O7	−177.9 (5)
C5—C6—C7—O3	−176.4 (4)	C17—O7—C16—C15	−108.1 (5)
C5—C6—C7—C2	1.3 (8)	C17—O7—C16—C11	73.3 (6)
C8—O3—C7—C6	−118.6 (5)	C12—C11—C16—C15	1.8 (7)
C8—O3—C7—C2	63.6 (7)	C10—C11—C16—C15	−177.0 (5)
C3—C2—C7—C6	−0.9 (8)	C12—C11—C16—O7	−179.7 (4)
C1—C2—C7—C6	176.7 (5)	C10—C11—C16—O7	1.5 (7)
C3—C2—C7—O3	176.7 (4)	C16—O7—C17—O8	−0.4 (7)
C1—C2—C7—O3	−5.7 (8)	C16—O7—C17—C18	180.0 (4)

Symmetry codes: (i) −*x*+2, −*y*+1, −*z*+1; (ii) −*x*+2, −*y*+2, −*z*+1.

### Hydrogen-bond geometry (Å, °)

**Table:**

*D*—H···*A*	*D*—H	H···*A*	*D*···*A*	*D*—H···*A*
O9—H2···O2^iii^	0.82	2.01	2.835 (7)	180.(1)
O9—H1···O5^iii^	0.82	1.93	2.758 (5)	179.(1)
C9—H92···O1^iv^	0.95	2.49	3.409 (9)	163
C15—H151···O4^ii^	0.95	2.51	3.221 (8)	132
C18—H181···O5^ii^	0.95	2.59	3.530 (9)	169

Symmetry codes: (iii) *x*+1, *y*, *z*; (iv) *x*−1, *y*, *z*; (ii) −*x*+2, −*y*+2, −*z*+1.
